# Preventive effect of polysaccharides from the large yellow croaker swim bladder on HCl/ethanol induced gastric injury in mice

**DOI:** 10.3892/etm.2014.1712

**Published:** 2014-05-14

**Authors:** SHAOCHENG CHEN, KAI ZHU, RUI WANG, XIN ZHAO

**Affiliations:** Department of Biological and Chemical Engineering, Chongqing University of Education, Chongqing 400067, P.R. China

**Keywords:** polysaccharide, large yellow croaker, gastric injury, cytokine, ICR mice

## Abstract

In the present study the preventive effect of polysaccharides from the large yellow croaker swim bladder (PLYCSB) on HCl/ethanol-induced gastric injury in ICR mice was investigated. A high dose of PLYCSB (50 mg/kg) was found to reduce the levels of the serum proinflammatory cytokines interleukin (IL)-1β, IL-6, IL-8, as well as increase the levels of IL-4 compared with those in mice treated with a low dose of PLYCSB (25 mg/kg) and control mice. The somatostatin and vasoactive intestinal peptide serum levels in PLYCSB-treated mice were higher compared with those in control mice, whilst motilin and substance P serum levels were lower compared with those in control mice. The extent of the gastric injury in the mice treated with PLYCSB was lower compared with that in the control mice; however, the results obtained for mice treated with a high dose of PLYCSB were similar to those for omeprazole-treated mice. In addition, the superoxide dismutase and glutathione peroxidase activities of PLYCSB-treated mice were higher compared with those of the control mice, and similar to those observed in normal and omeprazole-treated mice. Furthermore, PLYCSB-treated mice showed levels of nitric oxide and malondialdehyde that were similar to those in the normal group. Using PCR and western blot analysis, it was demonstrated that PLYCSB significantly inhibited inflammation in the tissues of the HCl/ethanol induced gastric injury mice by downregulating the expression of inducible nitric oxide synthase, cyclooxygenase-2, tumor necrosis factor-α and IL-1β. These results suggest that PLYCSB has an inhibitory effect against gastric injury that is comparable to that of the gastric injury drug omeprazole. Therefore, PLYCSB has the potential to be used as a natural therapeutic drug.

## Introduction

The swim bladder is an organ that is important for balance and polysaccharides account for as much as 10% of its weight. The large yellow croaker (*Larimichthys crocea*) is one of the main commercial fish in the coastal waters of China, and its swim bladder is rich in protein, microelements and vitamins. It is used in traditional Chinese medicine since it is considered to have curative effects against a number of different conditions, including amnesia, insomnia, dizziness, anepithymia and weakness after giving birth (1). A previous study has also suggested that the large yellow croaker swim bladder may serve to remove free radicals and protect against certain types of cancer (2). Polysaccharides are important functional materials. It has been shown that polysaccharides present in the swim bladder may accelerate wound healing, as well as prevent infection and thrombus formation (3). In addition, *in vivo* studies have demonstrated that polysaccharides from *Lentinus edodes* and spirulina seaweed serve to prevent and cure injury (4,5).

Gastric injury is damage to the stomach. It may occur due to chemical injury and may involve injury to the gastric mucosa. Ethanol promotes the rapid formation of lesions in the stomach due to an inflammatory reaction (6). Ethanol-induced gastric injury is associated with the loss of epithelial cells, mucosal edema and subepithelial hemorrhage (7). Therefore, in the present study HCl/ethanol was used for the chemical induction of gastric injury.

In the present study, the preventive effect of polysaccharides from the large yellow croaker swim bladder (PLYCSB) on gastric injury was investigated. The serum levels of inflammatory-associated cytokines were used to evaluate the preventive effect of PLYCSB on HCl/ethanol-induced gastric injury in ICR mice. In addition, gastric tissue histology was used to determine the preventive effects *in vivo*. Furthermore, the mRNA and protein expression levels of superoxide dismutase (SOD), glutathione peroxidase (GSH-Px), nitric oxide (NO) and malondialdehyde (MDA) in the tissues were analyzed in order to determine the preventive effect of PLYCSB.

## Materials and methods

### PLYCSB preparation

Wild Yellow Sea *Larimichthys crocea* were purchased in Shandong, China. The swim bladders from the *Larimichthys crocea* (1 kg) were freeze-dried and then crushed. Petroleum ether (3 l) was added to the swim bladder and reflux extraction was performed twice (1 h each time) at 60°C to remove the protein, and the residue was gathered following filtration. A total of 3 l absolute ethanol was then added and reflux extraction was performed for a further 3 h, and the residue without protein was filtered and gathered. Finally, 3 l water was added, the residue was extracted at 60°C for 2 h and the filtered liquid was collected. Crude polysaccharides from the large yellow croaker swim bladder were obtained following evaporation (8).

### Animals

Seven-week-old male ICR mice (n=50) were purchased from the Experimental Animal Center of Chongqing Medical University (Chongqing, China). The mice were maintained at 23±1°C with a relative humidity of 50±5% and a 12-h light/dark cycle. The mice had unlimited access to a standard mouse chow diet and water.

### Gastric injury experiment

The mice were divided into five groups (n=10 in each group). The normal group mice received no treatment during the experimental period. The control group mice received no treatment for the first 4 weeks. The PLYCSB group mice were orally administered either 25 or 50 mg/kg PLYCSB every day for 4 weeks. The mice of the omeprazole group (a drug cure comparator group) received a 25 mg/kg oral dose of omeprazole daily for 4 weeks. Then, after fasting for 24 h, the control and treatment groups were administered 1 ml HCl/ethanol (60% in 150 mM HCl) via esophageal intubation. After 1 h the mice were sacrificed using ether anesthesia. The stomachs were removed and 10 ml formalin (1%) was injected for 10 min to inflate the stomach and to fix the tissue walls, as well as to open the greater curvature (9). The

### of hemorrhagic lesions developed in the stomach was measured using a digital camera (D550; Canon, Tokyo, Japan) with a square grid

The images were analyzed using ImageJ software (National Institutes of Health, Bethesda, MD, USA) using the following formula: Gastric injury inhibitory rate (%) = (gastric injury area of control mice - gastric injury area of treated mice)/gastric injury area of control mice. The gastric secretion volume from each mouse was measured using a 10-ml measuring cylinder. The pH of the gastric juice was measured using a pH meter (SevenEasy pH meter; Mettler Toledo, Schwerzenbach, Switzerland) after being diluted 10-fold with distilled water. The experimental protocol was approved by the Animal Ethics Committee of Chongqing Medical University.

### Analysis of inflammation-associated cytokines in serum by enzyme-linked immunosorbent assay (ELISA)

For the serum cytokine assay, blood from the inferior vena cava was collected in a tube and centrifuged at 730 × g at 4°C for 10 min. The serum was aspirated and assayed as described below. The concentrations of inflammation-associated cytokines, interleukin (IL)-1β, IL-4, IL-6 and IL-8, in serum were measured using an ELISA in accordance with the manufacturer’s instructions (Biolegend, San Diego, CA, USA). Briefly, biotinylated antibody reagent was added to 96-well plates. The supernatants from the homogenized serum were then added and the plates were incubated at 37°C in CO_2_ for 2 h. The plates were washed with phosphate-buffered saline (PBS), streptavidin-horseradish peroxidase (HRP) solution was added and the plate was then incubated for a further 30 min at room temperature. The absorbance was measured at 450 nm using a microplate reader (iMark; Bio-Rad, Hercules, CA, USA) (10).

### Determination of MOT (motilin), SS (somatostatin), SP (substance P) and VIP (vasoactive intestinal peptide) levels in the serum

Blood was collected from the mice in a tube and centrifuged at 730 × g at 4°C for 10 min. The MOT, SS, SP and VIP levels in the serum were then determined using commercially available kits (Beijing Pu’er Weiye Bio-Technology Co., Ltd., Beijing, China).

### Determination of SOD, GSH-Px, NO and MDA levels

The gastric tissue was homogenized using a high-speed tissue homogenizer (T10; IKA-Werke GmbH & Co. KG, Staufen, Germany) at 730 × g at 4°C for 10 min. The SOD, GSH-Px, NO and MDA levels were then determined using commercially available kits (Nanjing Juli Institute of Biomedical Engineering, Nanjing, China).

### Analysis of the expression of inflammation-associated genes in gastric tissue using PCR

Total RNA from the gastric tissue cells was isolated using TRIzol^®^ reagent (Invitrogen Life Technologies, Carlsbad, CA, USA) in accordance with the manufacturer’s instructions. The RNA was digested with RNase-free DNase (Roche, Basel, Switzerland) for 15 min at 37°C and purified using an RNeasy kit (Qiagen, Hilden, Germany) in accordance with the manufacturer’s instructions. Total RNA (2 μg) was incubated at 37°C for l h with avian myeloblastosis virus reverse transcriptase (GE Healthcare, Little Chalfont, United Kingdom) with random hexanucleotides to form cDNA, in accordance with the manufacturer’s instructions. The following primers were used to specifically amplify the genes of interest: inducible nitric oxide synthase (iNOS) forward, 5′-AGA GAG ATC GGG TTC ACA-3′ and reverse, 5′-CAC AGA ACT GAG GGT ACA-3′; cyclooxygenase-2 (COX-2) forward, 5′-TTA AAA TGA GAT TGT CCG AA-3′ and reverse, 5′-AGA TCA CCT CTG CCT GAG TA-3′; tumor necrosis factor-α (TNF-α) forward, 5′-GAC CCT CAG ACT CAG ATC ATC CTT CT-3′ and reverse, 5′-ACG CTG GCT CAG CCA CTC-3′; and IL-1β forward, 5′-CTC CAT GAG CTT TGT ACA AGG-3′ and reverse, 5′-TGC TGA TGT ACC AGT TGG GG-3′. The internal control gene of GAPDH was amplified using the following primers: forward, 5′-CGG AGT CAA CGG ATT TGG TC-3′ and reverse, 5′-AGC CTT CTC CAT GGT CGT GA-3′. Amplification was performed in a thermal cycler (Eppendorf, Hamburg, Germany). The PCR products were separated on a 1.0% agarose gel and visualized using ethidium bromide staining (11).

### Protein extraction and western blot analysis of the gastric tissue

The total protein was obtained from the gastric tissue using Radio-Immunoprecipitation Assay buffer as previously described (11). The protein concentrations were determined using a Bio-Rad protein assay kit. For the western blot analysis, aliquots of the lysate containing 30–50 μg protein were separated using sodium dodecyl sulfate-polyacrylamide gel electrophoresis (SDS-PAGE) and then electrotransferred onto a nitrocellulose membrane (Schleicher and Schuell, Keene, NH, USA). The membranes were then subjected to immunoblot analysis and the proteins were visualized using an enhanced chemiluminescence (ECL) method (GE Healthcare). The cell lysates were separated using 12% SDS-PAGE, transferred onto a polyvinylidene fluoride membrane (GE Healthcare), blocked with 5% skimmed milk and then hybridized with primary antibodies (diluted 1:1,000). The antibodies against TNF-α, IL-1β, iNOS and COX-2 were obtained from Santa Cruz Biotechnology. The membranes were then incubated with the HRP-conjugated secondary antibodies (Santa Cruz Biotechnology) for 1 h at room temperature. The blots were washed 3 times with PBS-T and then developed using an electrochemiluminescence (ECL) reagent (Amersham Life Science, Arlington Heights, IL, USA).

### Statistical analysis

Data are presented as the mean ± standard deviation. Differences between the mean values between the groups were analyzed using a one-way analysis of variance with Duncan’s multiple range test. P<0.05 was considered to indicate a statistically significant difference. SAS version 9.1 (SAS Institute Inc., Cary, NC, USA) was used to conduct the statistical analyses.

## Results

### Gastric injury levels

**Figure 1:**
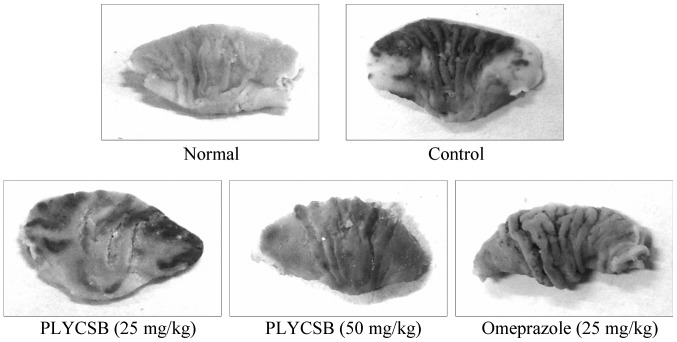
Stomachs of the mice treated with PLYCSB following the induction of gastric injury with HCl/ethanol. PLYCSB, polysaccharides of large yellow croaker swim bladder

Administration of PLYCSB to mice prior to the induction of gastritis was found to reduce gastric injury. The mice in the control group demonstrated a gastric injury area of 19.25±3.86 mm^2^. Treatment with 25 and 50 mg/kg PLYCSB resulted in a gastric injury inhibition index of 38.34 and 66.49%, respectively (Table I and Fig. 1). In particular, the greater level of protection against gastric injury was achieved with the higher dose of PLYCSB. The protective effect of 50 mg/kg PLYCSB was comparable with that observed for omeprazole (79.84%), which was used as the positive drug control.

### Gastric secretion volume and pH of the gastric juice

**Figure 2:**
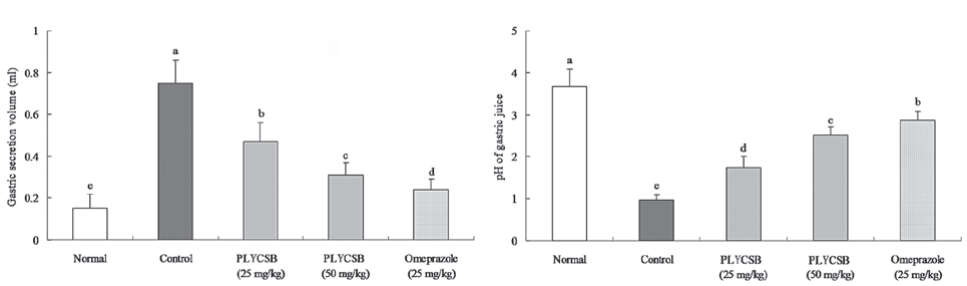
Gastric secretion volume of mice treated with PLYCSB following the induction of gastric injury with HCl/ethanol. Mean values with different letters over the bars are significantly different (P<0.05) according to Duncan’s multiple range test. PLYCSB, polysaccharides of large yellow croaker swim bladder.

The gastric secretion volume of normal mice was the lowest (0.15±0.07 ml) among all the groups (Fig. 2A). The volume was increased in the control mice (0.75±0.11 ml); however, the increase was attenuated following treatment with 25 mg/kg PLYCSB (0.47±0.09 ml), 50 mg/kg PLYCSB (0.31±0.06 ml) and omeprazole (0.24±0.05 ml). The pH values of the gastric juices were 3.68±0.42, 0.98±0.12, 1.74±0.27, 2.51±0.21 and 2.86±0.22 for the normal, control, 25 mg/kg PLYCSB, 50 mg/kg PLYCSB and omeprazole groups, respectively (Fig. 2B). These results demonstrate that there is a reduction in gastric secretion and an increase in gastric pH in mice treated with PLYCSB compared with those in the control mice.

### Effect of PLYCSB on serum levels of the cytokines IL-1β, IL-4, IL-6 and IL-8

The levels of IL-1β, IL-6 and IL-8 were lowest in the normal mice; however, in control mice these levels were significantly increased (Table II). The levels of IL-1β, IL-6 and IL-8 in mice treated with 25 and 50 mg/kg PLYCSB were lower compared with those in control mice. Furthermore, the mice treated with 50 mg/kg PLYCSB showed expression levels of IL-1β, IL-6 and IL-8 that were comparable with those in mice treated with omeprazole, and the expression levels were only slightly higher compared with those in normal mice. The level of IL-4 in all groups showed the opposite trend compared with that for the levels of IL-1β, IL-6 and IL-8. In the present study it was shown that the levels of IL-1β, IL-6 and IL-8 in the HCl/ethanol-induced gastric injury mice were markedly decreased, whilst the level of IL-4 was markedly increased following treatment with PLYCSB.

### Effect of PLYCSB on serum levels of MOT, SS, SP and VIP

The levels of MOT and SP were highest in the control mice, whilst the levels of SS and VIP were lowest in the control mice amongst all groups (Table III). In the PLYCSB-treated mice, the levels of MOT, SP, SS and VIP were significantly different from those in the control mice. The high dose of PLYCSB (50 mg/kg PLYCSB) and omeprazole treatment significantly attenuated the HCl/ethanol-induced changes in these levels, with results comparable with those in the normal mice.

### SOD, GSH-Px, NO and MDA levels in gastric tissue

The activities of SOD and GSH-Px in the gastric tissue of normal mice were higher compared with those in the other groups (Table IV). These activities were significantly decreased in the control mice. Mice treated with 50 mg/kg PLYCSB and omeprazole showed SOD activities similar to those in normal mice; however, the activity in mice treated with 25 mg/kg PLYCSB was decreased compared with that in 50 mg/kg PLYCSB-treated mice. The activity of GSH-Px in each group followed the same trend as the activity of SOD. The NO levels in each group of mice decreased in the order normal, omeprazole, 50 mg/kg PLYCSB, 25 mg/kg PLYCSB and control. The MDA levels of these groups showed the opposite trend from that observed for the NO levels.

### Effect of PLYCSB on the expression of inflammation-associated genes iNOS, COX-2, TNF-α and IL-1β

**Figure 3:**
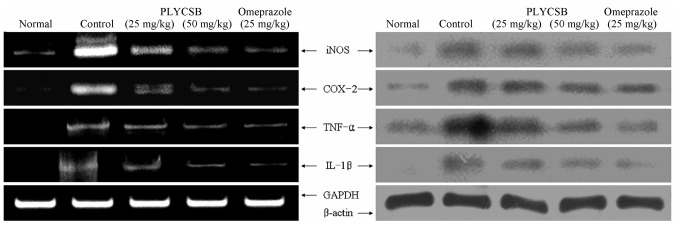
Effect of of PLYCSB on the mRNA and protein expression of iNOS, COX-2, TNF-α and IL-1β in mice with HCl/ethanol-induced gastric injury. PLYCSB, polysaccharides of large yellow croaker swim bladder; iNOS, inducible nitric oxide synthase; COX-2, cyclooxygenase-2; TNF-α, tumor necrosis factor-α; IL-1β, interleukin-1β.

The present study investigated whether the anti-inflammatory actions of PLYCSB were associated with an inhibition of inflammation-associated genes, specifically iNOS, COX-2, TNF-α and IL-1β. As shown in Fig. 3, the mRNA and protein expression levels of iNOS, COX-2, TNF-α and IL-1β were reduced in the gastric tissues treated with PLYCSB and omeprazole compared with those in the control tissues. PLYCSB and omeprazole significantly modulated the expression of genes associated with inflammation. Additionally, the mRNA and protein expression levels of these genes were decreased in the presence of the PLYCSB in a dose-dependent manner. These findings indicate that PLYCSB may help prevent gastric injury by increasing anti-inflammatory activities. In combination, these results showed that PLYCSB has a strong anti-inflammatory effect on gastric injury.

## Discussion

The swim bladder has been historically used in traditional Chinese medicine. The swim bladder has previously been shown to ameliorate different pathological conditions associated with inflammation, and it also been demonstrated to strengthen platelet function, capillary vessels and clotting factors (12). Swim bladders are mainly composed of polysaccharides; however, few studies have investigated the function of the polysaccharides from the swim bladder. In the present study, the preventive effect of PLYCSB on gastric injury was investigated for the first time, to the best of our knowledge. The results demonstrated that PLYCSB conferred the same level of protection against gastric injury as omeprazole, a drug used to treat gastritis.

A highly acidic environment in the stomach is an important marker of gastric injury. Gastric injury causes an increase in gastric secretion and acid output, resulting in a significantly decreased gastric pH (13). Mice treated with 50 mg/kg PLYCSB had decreased gastric secretion and a higher gastric pH compared with that in the control and low dose PLYCSB groups. This may explain why 50 mg/kg PLYCSB demonstrated a greater protective effect against gastric injury, compared with that of 25 mg/kg PLYCSB. In the present study, PLYCSB was found to exhibit a preventive effect on gastric injury by decreasing the levels of stomach acid.

The levels of serum cytokines, including IL-6 and IL-12, in patients with inflammatory diseases are elevated compared with those in healthy individuals (14). Cytokine receptors and the inflammatory cytokines IL-6 and IL-12 have a pathogenic role in gastric disease, and decreased expression levels of these indicate a preventive effect against gastric injury (15,16). IL-6 is an interleukin that functions as a proinflammatory and anti-inflammatory cytokine (17). T cells and macrophages secrete IL-6 to stimulate an immune response, particularly during tissue damage, which leads to inflammation. IL-6 is also involved in fighting infection (18). IL-4 has an important role in inflammation and wound repair and an increase in IL-4 secretion may aid the treatment of inflammatory disease (19). IL-8 is also associated with inflammation. Oxidative stress increases IL-8 secretion, which then causes the recruitment of inflammatory cells and induces an increase in oxidative stress mediators, making IL-8 a significant factor in localized inflammation (20).

MOT and SP are excitatory gastrointestinal hormones. The levels of MOT and SP increase following stimulation by gastric injury (21). Once MOT is stimulated, it causes the surplus secretion of gastric acid. An increase in gastric acid causes the inner part of the stomach to become too acidic, thereby compounding gastric injury (22). The results from the present study show that the levels of MOT and SP increase following treatment with HCl/ethanol. By contrast, SS and VIP are inhibitory gastrointestinal hormones, which are capable of inhibiting the secretion of gastric acid (21). It has been previously demonstrated that damaging the gastric mucosa results in the surplus secretion of gastric fluids and a reduction of the pH to a value lower than the normal value (23). The levels of SS and VIP were observed to increase following treatment with a high dose of PLYCSB compared with the levels in the control mice, which is likely to result in a reduction in gastric secretion volume and an increase in the pH of gastric juice. In the present study, the gastric secretion and pH of the gastric juice were in accordance with this.

Following gastric injury, the gastric tissue may be partially oxidized due to damage. SOD and GSH-Px are important antioxidants that reduce peroxide in the gastric tissue into less harmful or harmless substances, which is important in the recovery of gastric injury (24). Ethanol-induced gastric mucosal damage may involve the generation of oxygen-derived radicals, independent of the xanthine oxidase system. By acting as oxygen radical scavengers, SOD and GSH-Px provide significant gastroprotection (25). Tissue injury is caused by an imbalance between the damage of the gastric tissue and protective factors. NO serves to protect the gastric mucosa and keep the blood flowing smoothly. NO levels decrease significantly in patients suffering from gastric injury. In addition, NO has been demonstrated to be an effective component to guard against gastric injury (26). MDA is a marker of oxidative stress and it is generated in large amounts in the damaged areas of gastric tissue. Therefore, MDA can be used as an indicator of gastric injury (27). The results from the present study demonstrated that a higher concentration of PLYCSB decreases the degree of gastric injury.

iNOS, COX-2, TNF-α and IL-1β genes in the tissue may be used as biomarkers to monitor visceral damage. Following inflammatory stimuli, COX-2 and iNOS have been shown to induce deleterious effects in the stomach (28). iNOS and COX 2 in order to boost inflammatory responses in early stages of tissues injury (29). Inflammatory processes are mediated by multiple molecular mechanisms. Two of the most important are those associated with the production of iNOS and COX-2. The time courses by which inflammatory stimuli elicit iNOS and COX-2 protein synthesis are similar, which indicates that the two systems may interact with each other (30). The expression levels of TNF-α and IL-1β in patients with inflammatory diseases are higher compared with those in healthy individuals, and lower expression levels of TNF-α and IL-1β have been found to be indicative of improved anti-inflammatory effects (23). In the present study, it was demonstrated that PLYCSB significantly suppressed the expression of the inflammatory genes iNOS, COX-2, TNF-α and IL-1β. The levels of the protein products of these genes were also reduced.

In conclusion, the preventive effect of PLYCSB against gastric injury was evaluated in the present study using various *in vivo* experimental methods, including the use of a serum cytokine assay to analyze the levels of IL-1β, IL-4, IL-6 and IL-8; analysis of the serum levels of MOT, SS, SP and VIP; investigating the tissue levels of SOD, GSH-Px, NO and MDA, and using PCR and western blot analysis to determine the expression levels of inflammatory-associated genes and proteins, specifically iNOS, COX-2, TNF-α and IL-1β. Observation of the stomachs of mice in the different treatment groups revealed that PLYCSB had a preventive effect against HCl/ethanol-induced gastric injury, indicating that PLYCSB represents a potentially useful agent for the treatment or prevention of drug-induced gastric injury *in vivo*.

## Figures and Tables

**Table I:** Prevention of HCl/ethanol-induced gastric injury in ICR mice by treatment with PLYCSB.

	Rate of gastric injury inhibition	
	
Group	Gastric injury (mm^2^)	Inhibitory rate (%)
Normal	0.00±0.00^e^	100.00
Control	19.25±3.86^a^	0.00
PLYCSB (25 mg/kg)	11.87±2.66^b^	38.34
PLYCSB (50 mg/kg)	6.45±1.32^c^	66.49
Omeprazole (25 mg/kg)	3.88±0.98^d^	79.84

a–e Mean values with different letters in the same column are significantly different (P<0.05) according to Duncan’s multiple range test.

PLYCSB, polysaccharides of large yellow croaker swim bladder.

**Table II:** Serum IL-1β, IL-4, IL-6 and IL-8 cytokine levels of PLYCSB-treated mice with HCl/ethanol-induced gastric injury.

Group	IL-1β (pg/ml)	IL-4 (pg/ml)	IL-6 (pg/ml)	IL-8 (pg/ml)
Normal	44.82±3.62^e^	25.12±1.15^a^	32.71±3.82^e^	45.52±2.85^e^
Control	120.65±17.56^a^	10.65±1.34^e^	97.25±9.70^a^	91.34±1.92^a^
PLYCSB (25 mg/kg)	90.66±7.78^b^	15.24±0.92^d^	69.88±5.57^b^	73.18±2.02^b^
PLYCSB (50 mg/kg)	71.97±3.58^c^	18.98±0.61^c^	50.53±3.35^c^	62.15±1.71^c^
Omeprazole (25 mg/kg)	61.30±5.22^d^	21.12±0.82^b^	43.91±2.12^d^	57.63±1.32^d^

a–e Mean values with different letters in the same column are significantly different (P<0.05) according to Duncan’s multiple range test.

PLYCSB, polysaccharides of large yellow croaker swim bladder; IL, interleukin.

**Table III:** Serum MOT, SS, SP and VIP levels of PLYCSB-treated mice with HCl/ethanol-induced gastric injury.

Group	MOT (μg/l)	SS (μg/l)	SP (μg/l)	VIP (μg/l)
Normal	41.3±2.1^e^	115.6±10.7^a^	60.6±2.1^e^	97.7±3.4^a^
Control	93.6±4.1^a^	57.6±4.2^e^	118.3±2.6^a^	43.8±1.8^b^
PLYCSB (25 mg/kg)	67.3±5.7^b^	75.2±5.1^d^	83.6±2.2^b^	60.3±1.9^d^
PLYCSB (50 mg/kg)	56.1±3.2^c^	90.3±2.5^c^	72.6±2.8^c^	78.6±2.0^c^
Omeprazole (25 mg/kg)	51.2±2.0^d^	98.3±6.6^b^	67.3±2.4^d^	84.6±1.8^e^

a–e Mean values with different letters in the same column are significantly different (P<0.05) according to Duncan’s multiple range test.

PLYCSB, polysaccharides of large yellow croaker swim bladder; MOT, motilin; SS, somatostatin; SP, substance P; VIP, vasoactive intestinal peptide

**Table IV:** Tissue SOD, GSH-Px, NO and MDA levels in PLYCSB-treated mice with HCl/ethanol-induced gastric injury.

Group	SOD (kU/l)	GSH-Px (mmol/l)	NO (μmol/l)	MDA (μmol/l)
Normal	336.12±38.92^a^	3.92±0.37^a^	13.74±1.55^a^	12.87±1.79^e^
Control	222.35±35.11^e^	2.11±0.28^e^	2.35±0.45^e^	63.11±4.56^a^
PLYCSB (25 mg/kg)	262.91±27.79^d^	2.79±0.30^d^	6.11±1.08^d^	41.36±3.87^b^
PLYCSB (50 mg/kg)	291.30±17.28^c^	3.41±0.22^c^	10.36±1.05^c^	28.71±1.56^c^
Omeprazole (25 mg/kg)	308.76±22.35^b^	3.61±0.12^b^	11.85±0.42^b^	19.78±3.54^d^

a–e Mean values with different letters in the same column are significantly different (P<0.05) according to Duncan’s multiple range test.

PLYCSB, polysaccharides of large yellow croaker swim bladder; SOD, superoxide dismutase; GSH-Px, glutathione peroxidase; NO, nitrogen oxide; MDA, malondialdehyde.

## References

[1] Jian JC, Wu ZH (2003). Effects of traditional Chinese medicine on nonspecific immunity and disease resistance of large yellow croaker, *Pseudosciaena crocea* (Richardson). Aquaculture.

[2] Li C, Yao CL (2013). Molecular and expression characterizations of interleukin-8 gene in large yellow croaker (*Larimichthys crocea*). Fish Shellfish Immunol.

[3] Liu S, Yu B (2009). Peptides from variegated carp (*Aristichthys nobilis*) swim bladder: Fermentation production and assessment of antioxidant properties. Food Sci.

[4] Yu ZH, Yin LH, Yang Q, Liu Y (2009). Effect of *Lentinus edodes* polysaccharide on oxidative stress, immunity activity and oral ulceration of rats stimulated by phenol. Carbohydr Polym.

[5] Laurienzo P (2010). Marine polysaccharides in pharmaceutical applications: an overview. Mar Drugs.

[6] Szabo S, Trier JS, Brown A, Schnoor J (1985). Early vascular injury and increased vascular permeability in gastric mucosal injury caused by ethanol in the rat. Gastroenterology.

[7] Medeiros JV, Gadelha GG, Lima SJ, Garcia JA, Soares PM, Santos AA, Brito GA, Ribeiro RA, Souza MH (2008). Role of the NO/cGMP/K(ATP) pathway in the protective effects of sildenafil against ethanol-induced gastric damage in rats. Br J Pharmacol.

[8] Yan C, Yan X (2008). Study on extraction of *Lycium barbarum* polysaccharides by different methods and their antioxidant effects in vitro. Food Sci.

[9] Ramirez RO, Roa CC (2003). The gastroprotective effect of tannins extracted from duhat (*Syzygium cumini* Skeels) bark on HCl/ethanol induced gastric mucosal injury in Sprague-Dawley rats. Clin Hemorheol Microcirc.

[10] Wang Q, Zhao X, Qian Y, Wang R (2013). In vitro antioxidative activity of yellow tea and its in vivo preventive effect on gastric injury. Exp Ther Med.

[11] Zhao X, Kim SY, Park KY (2013). Bamboo salt has in vitro anticancer activity in HCT-116 cells and exerts anti-metastatic effects in vivo. J Med Food.

[12] Cao H, Tian XL, Liu X (2009). Study on molecular identification and pharmacology of hemostasis action for isinglass. Zhongguo Shipin Xuebao.

[13] Ligumsky M, Sestieri M, Okon E, Ginsburg I (1995). Antioxidants inhibit ethanol-induced gastric injury in the rat. Role of manganese, glycine, and carotene. Scand J Gastroenterol.

[14] Gratacós J, Collado A, Filella X, Sanmartí R, Cañete J, Llena J, Molina R, Ballesta A, Muñoz-Gómez J (1994). Serum cytokines (IL-6, TNF-alpha, IL-1 beta and IFN-gamma) in ankylosing spondylitis: a close correlation between serum IL-6 and disease activity and severity. Br J Rheumatol.

[15] Fox JG, Beck P, Dangler CA, Whary MT, Wang TC, Shi NH, Nagler-Anderson C (2000). Concurrent enteric helminth infection modulates inflammation and gastric immune responses and reduces helicobacter-induced gastric atrophy. Nat Med.

[16] D’Elios MM, Manghetti M, De Carli M, Costa F, Baldari CT, Burroni D, Telford JL, Romagnani S, Del Prete G (1997). T helper 1 effector cells specific for *Helicobacter pylori* in the gastric antrum of patients with peptic ulcer disease. J Immunol.

[17] Ferguson-Smith AC, Chen YF, Newman MS, May LT, Sehgal PB, Ruddle FH (1988). Regional localization of the interferon-beta 2/B-cell stimulatory factor 2/hepatocyte stimulating factor gene to human chromosome 7p15-p21. Genomics.

[18] van der Poll T, Keogh CV, Guirao X, Buurman WA, Kopf M, Lowry SF (1997). Interleukin-6 gene-deficient mice show impaired defense against pneumococcal pneumonia. J Infect Dis.

[19] Maeda S, Yanagihara Y (2001). Inflammatory cytokines (IL-4, IL-5 and IL-13). Nippon Rinsho.

[20] Vlahopoulos S, Boldogh I, Casola A, Brasier AR (1999). Nuclear factor-kappaB-dependent induction of interleukin-8 gene expression by tumor necrosis factor alpha: evidence for an antioxidant sensitive activating pathway distinct from nuclear translocation. Blood.

[21] Wang HY, Liu YM, Li HY, Feng QJ, Guo JY, Niu X (2011). Effects of oils in *Alpinia officinarum* Hance on serum motilin, somatostatin, substance P, vasoactive intestinal peptide in gastrelcosis mice model. Chinese J Exp Tradit Med Formulae.

[22] Zhang SR, Shao JY, Yu YW (1984). The protective effects of furazolidone and some commonly used antiulcer drugs on several gastric ulcer models in rats. Yao Xue Xue Bao.

[23] Zhao X, Wang Q, Qian Y, Song JL (2013). Ilex kudingcha C.J. Tseng (Kudingcha) prevents HCl/ethanol-induced gastric injury in Sprague-Dawley rats. Mol Med Rep.

[24] Qu CY, Li DG, Wang YQ, Chen MM (2008). Effect of chitosan on the serum levels of MDA, SOD, and GSH-Px in rats with gastric ulcer. Shanghai Med Pharm J.

[25] Ligumsky M, Sestieri M, Okon E, Ginsburg I (1995). Antioxidants inhibit ethanol-induced gastric injury in the rat: Role of manganese, glycine, and carotene. Scand J Gastroenterol.

[26] Cheng HH, AXR (2010). Change of serum nitrogen monoxidum and nitrogen monoxidum synthase levels in patients with peptic ulcer in high altitude region. Lab Med.

[27] Wang G, Tu ZL, Chen L, Yuan SH, Yang GY (2009). Mechanism of antiulcer effects of Jinguolan. Herald Med.

[28] Li HL, Sun BZ, Ma FC (2004). Expression of COX-2, iNOS, p53 and Ki-67 in gastric mucosa-associated lymphoid tissue lymphoma. World J Gastroenterol.

[29] Hayden MS, Ghosh S (2004). Signaling to NF-kappaB. Gene Dev.

[30] Kim SF, Huri DA, Snyder SH (2005). Inducible nitric oxide synthase binds, S-nitrosylates, and activates cyclooxygenase-2. Science.

